# Philadelphia Medical Society

**Published:** 1841-11-27

**Authors:** 


### PROCEEDINGS OF SOCIETIES.

### PHILADELPHIA MEDICAL SOCIETY.

*Session of Nov. 13th*, 1841.—Professor Robert M. Huston introduced the discussion of this evening by a series of verbal remarks on the subject of vaccination as an antidote to the contagion of small pox, and many interesting facts were elicited from Drs. Brewer, Bryan, B. Horner Coates, Reynell Coates, Griscom,

Kite, Parrish, Staples, Vandyke, jr., Warrington, and Wiltbank.

The remarks of the lecturer were principally designed to call out the opinion of the profession, and as comments, upon the question which has agitated the public mind since the appearance of the varioloid epidemic, and which is more important at the present moment in consequence of the existing epidemic tendency to small pox in this country. Dr. Huston fully believed, and amply illustrated, the permanent protective influence of vaccination, acknowledging, at the same time, that the perfect security supposed to result from it in the days of Jenner, is not now so generally felt, and that neither the natural or artificial production of true small pox, nor the proper kine pox infection, has proved to be an absolute protection in every case. He quoted a recent instance occurring in the practice of a respectable medical gentleman of this city, which had passed twice through the regular course of well-marked, natural, confluent small pox, with an interval of only six weeks between the two attacks. Two other cases of the recurrence of the modified disease occurring at very short intervals after the first regular attack, were mentioned by other practitioners during the discussion.

Dr. H. is of opinion that the modified disease is now more rife than it was at the time when the operation of vaccination first became general, but that unmodified small pox never occurs after successful vaccination. He inferred that the occurrence of varioloid eruption was rare under such circumstances, from the fact that in his long and extensive practice among children, but three instances had occurred in those on whom he had operated himself, and who continued subject to his inspection in after life: he being cautious never to vaccinate a person not in good health—and delaying the operation, even in those in danger of exposure to contagious miasm, rather than run the risk of an imperfect result, which might permanently prevent the beneficial tendency of future and better timed vaccination.

The general frequency of such occurrences in general practice he appeared to attribute mainly to the operation being so very frequently performed under constitutional conditions of the patient capable of seriously modifying the effect of the vaccination, and thus diminishing or destroying the preventive power of the disease.

In commenting upon this part of the lecture, Dr. B. H. Coates expressed his decided conviction that a partially successful vaccination might give partial protection against small pox, and lead to the occurrence of mild instead of severe disease from future exposure to the action of the virus.

Dr. Huston inquired what had been the experience of others as to the regularity of the career in the common mode of vaccination from the scab which might have been modified by the transmission of the virus through some hundreds of constitutions, each capable of influencing, to some extent, the character of the disease. His own experience, after reference to the statistics of the age of Jenner, and some experiments performed by himself, or by his medical acquaintance, on matter but a few degrees removed from the cow, led him to believe that the scab rarely adhered for so many days when the operation was performed—as is the almost universal custom in this country—with scabs from those to whom the virus had been transmitted through a great number of individuals, as when the more recent matter was employed. He appeared to be convinced that the duration of the disease was shortened by the constitutional modification of the matter employed, and inferred that this circumstance enhanced the liability to varioloid so frequently exemplified of late years.

This query elicited replies from most of the gentlemen who took part in the debate; but very few accurately recorded observations were quoted. The general impression seemed to be that the termination of the disease, or, rather, the falling of the scab, took place some days earlier now than in the days of Jenner.

Dr. Huston mentioned several striking cases of the powerful protective influence of vaccination under all the disadvantages—if such be acknowledged to exist—resulting from the frequent transfer of the virus.

Some years ago he delivered a female, the mother of nine children, who had all been vaccinated. The family in an adjoining house were labouring under the small pox. Dr. H. vaccinated the infant on the second day. The nurse was deeply pitted with small-pox, and boasted of her security from all attack. Yet she contracted the disease, and was conveyed to the Bush-hill Hospital. The infant escaped the infection.

In another case, very recently, a lady met an acquaintance in the street, directly from a house in which small pox existed at the time. Alarmed, she sent for Dr. H. to vaccinate her nursing infant. The infant took the vaccine, and, on the ninth day from the insertion of the vaccine matter in its arm, distinct, and well-marked small pox appeared on its mother. The child continued to draw nourishment from her breasts, but without suffering at all from the variolus infection.

During the latter part of the debate, Dr. R. Coates referred to a case occurring in the practice of his brother, and by him narrated in a former discussion before the College of Physicians, in which instance, an infant was vaccinated several days after exposure to the contagion of small pox. The fever and eruption of the latter disease appeared, continued for some days, delaying the access of the vaccine inflammation, then faded and gave place to the vaccine pustule, which afterwards pursued its regular course.

Dr. Huston mentioned several other very striking cases in proof of the protection afforded by vaccination, and the number was increased by the various gentlemen who took part in the debate. He stated that he never performed vaccination in infants, except upon emergencies, before the third or fourth month, and commented upon the impropriety of attempting it while there exist eruptions, sores behind the ears, summer complaint or dentition; dwelling particularly on the general exemption of children from the usual infantile diseases during the continuance of the irritation of the last named process.

The general tendency of the remarks of the lecturer was somewhat in opposition to the idea that revaccination, in cases where the operation had been originally well performed and properly watched, is a necessary precaution.

Dr. Reynell Coates inclined to the opinion of the lecturer on this subject, and commented upon the culpable carelessness of many practitioners in these respects. He dwelt strongly upon the severe effects sometimes resulting from the process of revaccination, such as erysipelatous and absorbent inflammation, stating that the virus, though harmless to many, always acted as a severe poison to others. He had tried it frequently upon himself and several of his immediate family, and generally with severe consequences.

It was inferable from the observations of Dr. B. Horner Coates, that he was at least doubtful on this subject. Several other gentlemen spoke to this point, but it did not appear that a very general or decided opinion upon it prevailed. Dr. Huston remarked upon the existence of idiosyncracies, which, by giving individuals peculiar exemption might confuse the calculation. Dr. B. H. Coates considered the age of the patient as a cause of diminished liability, and spoke of the acknowledged infrequency of small pox among old persons, and, particularly old white nurses, who should be preferred, on that account, for attendance on the sick. Dr. Warrington inquired if any member had ever seen a second vaccination result in a regular course of disease, and no case was adduced. Dr. B. H. Coates referred to a series of observations by Hufeland, in which that author was induced to conclude that the susceptibility to the reimpression of the virus was increased gradually for a number of years after vaccination. Dr. Wiltbank gave several cases of repeated attacks of varioloid after vaccination. Dr. Vandyke, Jr., Brewer, and others, related cases in which vaccination had produced no effect whatever.

Dr. Bryan quoted the opinion of the President of the Royal Institution in London in opposition to the idea that the vaccine virus became modified by frequent transmission. He gave his own experience in favour of the idea that the effect of virus but a few times removed from the cow was productive of more constitutional disturbance, and severer inflammation. His experience would have been more extensive had not the matter brought over by him from England between glasses, been vitiated by dampness on the voyage, which foreclosed his earlier experiments. This elicited the remark from Dr. Reynell Coates, that he had found the best mode of preserving the purity of vaccine matter for a long time to consist in enveloping the paper containing the scab in waxed bougie cloth, folded and warmed, so as to cause the folds to adhere, and exclude both air and water. Dr. Warrington had made and witnessed many trials with matter but a few times removed from the cow, and found its effect so severe as to dread its employment in children. The pustule is deeper and the scab more black. Dr. Parrish tried it but once, and was fearful of repeating the experiment from what he had seen and heard of the inflammation. Dr. W. remarked that the vaccine pustule was at this time rare in England, so that it was difficult to procure the original matter. He did not credit the diminished certainty resulting from the frequent transmissions of the virus through successive individuals. He stated that the effects of the more recent matter had rendered Dr. Samuel Jackson (late of Northumberland,) fearful of continuing its use. Some portions of scab but few degrees removed from the cow were produced and distributed among the members, for the purpose of eliciting practical tests in different parts of the United States. A member remarked that the pustule on the cow had not been discovered in this country. Dr. Reynell Coates corrected this error, stating that he had met with individuals who had seen it, and was under the impression, though not perfectly sure, that Dr. Richard Harlan, of this city, had experimented with matter of American origin.

In answer to a question from the last named speaker, in relation to the supposed identity of cow and small pox, Dr. Vandyke, Jr., stated that he had endeavoured, in four instances, without success, to innoculate the cow with the small pox in the West Indies. One of the cows had a small "fester" at the spot wounded, but no proper pustule of any kind occurred in either of them.

Dr. Reynell Coates remarked, that one cause of the failure of attempts to vaccinate, which often perplexes practitioners, and delays the security of the patient, is the habit of operating by simple puncture; the point of the lancet being wiped clean in elevating the cuticle, the virus washed out by the flow of blood which guards the living surfaces with a coagulum, or particles of matter arrested in contact with the inanimate epidermis alone. He stated that, by the ordinary method, the innoculation fails something like once in every three trials; and recommended the following process, which had been successful in every instance in his hands. Prepare the fragment of the scab, cut fine upon a plate of glass, or the handle of a silver teaspoon, previously moistened by a single drop of water, and the quantity still further reduced by contact with the end of the finger. Rub the fragments into a paste with the water. Then, with the heel of the lancet, abrade the spot chosen for the inocculation until the cuticle is nearly removed, and the blood upon the point of starting. Next, spread the pasty virus upon the surface, moistening it a little more, if necessary, by dipping the point of the lancet in water, and proceed to make a number of slight incisions through the remaining cuticle until traces of blood are visible, without producing actual hæmorrhage. Cross these incisions by others at a right angle, by a process like that termed "hatching" by draftsmen, and allow the paste to become dry upon the spot.

Dr. B. Horner Coates preferred positive bleeding from the spot in the first instance, and afterwards preparing the virus, as he had found the moistened matter to become very rapidly diminished in energy by the action of the air. He referred to several authorities and series of observations to prove that, though the varioloid occurred less frequently after natural or innoculated small pox, it presents greater gravity of character in such cases than in those which followed the kine pox innoculation. He stated that his recollection of the observations of others impressed him with the belief that the varioloid epidemics in America, where the scab is almost universally employed, had not been more severe than those occurring in England and France, where resort was usually had to the matter direct from the pustule. He terminated his remarks with an expostulation against the unguarded expressions of doubt on the part of many medical men, which were calculated improperly to shake the faith of the public in the blessings of vaccination.

